# Insights into Molecular Profiles, Resistance Patterns, and Virulence Traits of Staphylococci from Companion Dogs in Angola

**DOI:** 10.3390/ani15071043

**Published:** 2025-04-04

**Authors:** Romay Coragem da Costa, Francisca Guerra Cunha, Raquel Abreu, Gonçalo Pereira, Catarina Geraldes, Eva Cunha, Lélia Chambel, Manuela Oliveira

**Affiliations:** 1 CIISA—Centre for Interdisciplinary Research in Animal Health, Faculty of Veterinary Medicine, University of Lisbon, Avenida da Universidade Técnica, 1300-477 Lisboa, Portugal; rccosta@fmv.ulisboa.pt (R.C.d.C.); francisca.guerra.cunha@gmail.com (F.G.C.); rmsilva@fmv.ulisboa.pt (R.A.); goncalopereira@fmv.ulisboa.pt (G.P.); cgeraldes@fmv.ulisboa.pt (C.G.); moliveira@fmv.ulisboa.pt (M.O.); 2 Associate Laboratory for Animal and Veterinary Sciences (AL4AnimalS), 1300-477 Lisboa, Portugal; 3 Department of Animal Health, Faculty of Veterinary Medicine, University José Eduardo dos Santos, Huambo P.O. Box 2458, Angola; 4 BioISI—Biosystems and Integrative Sciences Institute, Faculty of Sciences, University of Lisbon, 1749-016 Lisboa, Portugal; lmchambel@ciencias.ulisboa.pt; 5 cE3c—Centre for Ecology, Evolution and Environmental Changes & CHANGE—Global Change and Sustainability Institute, 1749-016 Lisboa, Portugal

**Keywords:** Angola, antimicrobial resistance, dogs, staphylococci, virulence

## Abstract

Staphylococci are prevalent in dogs’ microbiota, with commensal strains being able to exhibit resistance and virulence traits, complicating secondary infection management. As antimicrobial resistance is a global threat, particularly in middle-income countries like Angola, surveillance of resistant bacteria is crucial. We analyzed the prevalence, antimicrobial resistance, and virulence profiles of staphylococci from dogs in Angola. Isolates were identified using VITEK^®^ 2 Compact (bioMérieux© SA, Marcy l’Etoile, France), and their genetic diversity was assessed via PCR fingerprinting. Isolates’ susceptibility to relevant antimicrobials was determined by disk diffusion, and their virulence profiles were evaluated using plaque assays. The relationship between antibiotic resistance and animal-related factors was also assessed by statistical analysis. Isolates were identified as *Mammaliicoccus sciuri* (former *Staphylococcus sciuri*, 38%), *Staphylococcus xylosus* (30%), *Staphylococcus equorum* (13%), *Mammaliicoccus vitulinus* (former *Staphylococcus vitulinus*, 7%), *Mammaliicoccus lentus* (former *Staphylococcus lentus*, 5%), *Staphylococcus aureus* (2%), and *Staphylococcus* spp. (5%). Of these, 86% were resistant to at least one of the antimicrobials tested, and 30% were classified as multidrug-resistant, being more common in females, dogs with clinical signs of disease, and vaccinated animals. Moreover, 93% of the isolates were able to produce biofilm, 46% could produce lecithinase and gelatinase, and 23% could produce hemolysins. Companion dogs from Angola can carry resistant staphylococci able to express several virulence factors, potentially representing a One Health risk.

## Simple Summary

Antimicrobial resistance is a global threat, particularly in middle-income countries like Angola, where there is limited information on this issue. Therefore, resistant bacteria monitoring is crucial. This study analyzed the prevalence, antimicrobial resistance, and virulence profiles of staphylococci of dogs from Angola. Several staphylococci were identified, with *Mammaliicoccus sciuri* and *Staphylococcus xylosus* being the most common species. Most isolates were resistant to at least one antimicrobial, and 30% were classified as multidrug-resistant, which were more frequent in females, sick dogs, and vaccinated animals. In addition, 93% of the isolates were able to produce biofilm, 46% could produce lecithinase and gelatinase, and 23% could produce hemolysins. The results indicate that in Angola pet dogs can be reservoirs of resistant staphylococci, posing a risk to public health.

## 1. Introduction

Staphylococcaceae is a diverse group of bacteria that includes both commensal and pathogenic species, such as *Staphylococcus* spp. and *Mammaliicoccus* spp. [1,2]. These Gram-positive bacteria are frequently found in the resident or transient microbiota of the skin and nasal mucous membranes of humans and animals, including dogs. Bacteria from these genera can also cause mild-to-severe infections, including nosocomial infections, which demonstrates their potential impact on public health [3,4]. In fact, staphylococcal infections are of special concern, considering their high levels of resistance to penicillins and other commonly used antimicrobials, which can reach values of up to 90% [5].

Despite staphylococci’s importance in human and veterinary medicine, little is known about the relevance of these bacteria in veterinary medicine in low- and middle-income (LMI) countries, including Angola [3]. In this country, the available information focuses only on isolates of human origin, with reports describing the colonization by Methicillin-resistant *Staphylococcus aureus* (MRSA) of children treated in the emergency and outpatient services of a pediatric hospital in Luanda [6] as well as of adult patients and healthcare professionals [7].

In contrast, there is no available information on the resistance and virulence profiles of staphylococci from companion animals in Angola, which is of major relevance since some studies have suggested that about 4% of antimicrobial resistance problems in humans may be associated with animals [8]. In fact, a study by Morris et al. [9] described the isolation of the same bacterial strains, including MRSA and methicillin-resistant *Staphylococcus pseudintermedius* (MRSP) strains, from humans and their pets. Likewise, a study from Australia suggested that MRSA carried by dogs were originated from human hosts [10].

Since Angola is highly vulnerable to infectious disease outbreaks, which endanger populations’ health, preventing the spread of resistant and virulent staphylococci strains between animals and humans is essential for safeguarding public health [6], especially considering that the genus *Staphylococcus* is included in the WHO Bacterial Priority Pathogens List [11]. Moreover, Angola does not have a national action plan aimed at controlling antimicrobial resistance (AMR) spread. This plan would be of utmost relevance to break the AMR transmission cycle and reduce mortality associated with diseases caused by bacterial species resistant to conventional antimicrobials [3].

Considering not only the role of animals in AMR dissemination in LMI countries [12] but also the relevance of resistant staphylococci in the context of One Health, this study aimed to evaluate the presence and AMR and virulence profiles of staphylococci from dogs in Angola, representing the first report characterizing antimicrobial-resistant staphylococci from dogs in this country.

## 2. Materials and Methods

### 2.1. Sample Collection

AMIES swab (VWR^®^, Leuven, Belgium) samples were collected from the skin and oral and nasal cavities of healthy and sick dogs presented for routine clinical consultations at a veterinary clinic in Huambo province of south-central Angola during March (wet season) and August (dry season) of 2022. The dogs selected for this study were not subjected to any antimicrobial treatment for at least two weeks before sampling, which was performed regardless of each animal’s age, breed, or sex.

It was possible to obtain a total of 96 samples from 32 dogs (skin n = 32, oral cavity n = 32, nasal cavity n = 32), which were kept refrigerated until transport to the Bacteriology Laboratory of the Faculty of Veterinary Medicine of the University of Lisbon in Portugal, where they were further processed. Information regarding the animals was also registered, including each dog’s age, sex, breed, neutered or intact status, outdoor access, presence or absence of disease, presumptive diagnosis, previous therapeutic protocols such as administration of antimicrobials, antiparasitic and anti-inflammatory drugs, previous surgeries, and vaccination schedule.

All animals were cared for according to the rules given by the current EU (Directive 2010/63/EC) and Portuguese (DL 113/2013) legislation by the competent authority in Portugal (https://www.dgav.pt/animais, accessed on 14 December 2024) and by the Regulation of the Animal Health Law of Angola (DL 70/08). Only noninvasive samples were collected during routine procedures with the consent of the owners, and no ethical approval was needed. Trained veterinarians obtained all samples while following standard routine procedures. No animal experiments were performed. Verbal informed consent was obtained from all owners, with all necessary information about the study being provided before obtaining participants’ consent.

### 2.2. Staphylococci Isolation

Swab samples were inoculated on Mannitol Salt agar (MSA, Scharlau^®^, Barcelona, Spain) plates incubated at 37 °C for 24 h. Afterward, single colonies were randomly selected from each culture, with a maximum of four colonies per plate, which were further isolated in pure cultures on MSA.

Selected isolates were characterized by Gram staining and catalase, coagulase, and mannitol fermentation. The isolates presumptively identified as staphylococci were kept at −20 °C in Buffered Peptone Water (BPW) (VWR^®^, Leuven, Belgium) with 20% glycerol (VWR^®^, Leuven, Belgium).

### 2.3. DNA Isolation

DNA from all the isolates and from the reference strain *Staphylococcus aureus* ATCC^®^ 25923™(Manassas, VA, USA) was obtained with the boiling method as described by Švec et al. [13]. The DNA’s purity and concentration were determined using a NanoDrop™ Spectrophotometer (ThermoFisher Scientific^®^, Waltham, MA, USA).

### 2.4. PCR Fingerprinting

A protocol adapted from Semedo-Lemsaddek et al. [14] was performed with the aim of isolates’ fingerprinting. Briefly, 2 μL of a suspension containing 100 ng of DNA was added to 23 μL of a PCR mixture containing 2 μM of the primer (GTG)_5_, 0.2 μM of each dNTP, 0.06 U of *Taq* polymerase (Invitrogen^®^, Waltham, MA, USA), 1×reaction buffer, and 3 μM MgCl_2_. Negative controls composed of PCR mixture and water were included in all reactions, and 10% of replicates were also analyzed. PCR amplification was performed using a thermocycler (VWR^®^ XT96, Wurzburg, Germany) and the following conditions: initial denaturation (94 °C for 4 min); 40 cycles of denaturation (94 °C for 1 min), annealing (40 °C for 2 min), and extension (72 °C for 2 min); and final extension (72 °C for 10 min). PCR products were visualized through electrophoresis on 1.5% agarose gel in 1× TBE buffer (NZYTech, Lisbon, Portugal), supplemented with Green Safe (NZYTech, Lisbon, Portugal), subjected to 90 V for 1 h 10 min, and photographed using the ChemiDocMP System Community (Bio-Rad Laboratories, Inc., Hercules, CA, USA). The molecular profiles obtained were analyzed using BioNumerics^®^ 6.6 (Applied Maths, Kortrijk, Belgium). All isolates were compared to each other based on dendrograms obtained from densitometric analysis of the molecular profiles using Pearson’s correlation coefficient (without optimization parameters) and the UPGMA agglomeration method.

### 2.5. Isolate Identification

The representative isolates selected based on their PCR fingerprinting profiles were identified using the VITEK^®^ 2 Compact and VITEK^®^ 2 GP ID cards (bioMérieux© SA, Marcy l’Etoile, France) according to the manufacturer’s instructions.

### 2.6. Isolates’ Antimicrobial Resistance Profile

The resistance profile of the representative isolates was determined using the disk diffusion method according to the Clinical and Laboratory Standards Institute (CLSI) guidelines [15,16,17] using 10 different antimicrobial compounds from diverse classes relevant to both veterinary and human medicine, including β-lactams [penicillins (ampicillin (AMP),10 μg), penicillins in combination with a β-lactamase inhibitor (amoxicillin-clavulanate (AMC), 30 μg), cephalosporins (cefoxitin (FOX), 30 μg), carbapenems (meropenem (MEM), 10 μg)], fluoroquinolones (enrofloxacin (ENR), 5 μg), tetracyclines (doxycycline (DO), 30 μg), aminoglycosides (gentamicin (CN), 10 μg), oxazolidinones (linezolid (LZD), 30 μg), folate antagonists (trimethoprim/sulfamethoxazole (SXT), 25 μg), and glycopeptides (vancomycin (VA), 30 μg) (Oxoid™, Hampshire, UK). The reference strain *Staphylococcus aureus* ATCC^®^ 25923™ (Manassas, VA, USA) was also tested as a quality control [17], as well as a 10% replica of randomly selected isolates [17].

Vancomycin resistance was assessed due to its public health importance, and corresponding results were evaluated using vancomycin breakpoints from CLSI M31-A3 [15].

Isolates phenotypically resistant to cefoxitin were classified as methicillin-resistant staphylococci (MRS) [17]. The categorization of the isolates as multidrug-resistant followed the criteria of Magiorakos et al. [18].

### 2.7. Isolates’ Virulence Profile

The phenotypic virulence profile of the isolates was determined as previously described [19] by assessing their ability to produce 4 different enzymes and biofilm using specific media. Briefly, hemolysins’ production was assessed using Columbia Agar plates supplemented with 5% sheep blood (bioMérieux©, Marcy l’Etoile, France), using *S. aureus* ATCC^®^ 25923^TM^ as a positive control and *Enterococcus faecium* CCUG^®^ 36804 as a negative control. Biofilm production was determined using Congo Red Agar plates, using *Escherichia coli* ATCC^®^ 25922™ and *E. faecalis* ATTC^®^ 29212™ as negative and positive controls, respectively. Lecithinase activity was tested using Tryptic Soy Agar supplemented with 10% egg yolk (VWR^®^, Leuven, Belgium), using *Pseudomonas aeruginosa* ATCC^®^ 27853™ as a positive control and *E. coli* ATCC^®^ 25922™ as a negative control. DNAse production was evaluated using DNAse Agar (Remel™, San Diego, CA, USA) supplemented with 0.01% Toluidine Blue (Merck, Darmstadt, Germany), using *Aeromonas hydrophila* ATCC^®^ 7966™ and *E. coli* ATCC^®^ 25922™ as positive and negative controls, respectively. Finally, gelatinase activity was determined using Nutrient Gelatin medium (Oxoid™, Basingstoke, UK), using *E. coli* ATCC^®^ 25922™ and *P. aeruginosa* Z25.1 (clinical isolate from a diabetic foot infection) as negative and positive controls, respectively [19].

In each assay, a 10% replica was also evaluated.

### 2.8. Statistical Analysis

The multiple antimicrobial resistance index (MAR index) and virulence index (V. index) values were determined for all isolates, according to Matos et al. [12], as follows:

$$MAR\;index=\frac{no.antimicrobials\;to\;which\;isolates\;were\;resistant}{no.antimicrobials\;tested}$$

$$V.index=\frac{no.positive\;virulence\;factors}{no.virulence\;factors\;tested}$$

Descriptive statistical analysis was performed using Microsoft© Office Excel 365 (Microsoft Corporation, Redmond, WA, USA). Further statistical analysis was performed using SAS 9.4 (SAS Institute Inc., Cary, NC, USA).

The relationship between isolates’ resistance to each antibiotic and the variables’ sampling season, sample origin, and each animal’s sex, presence or absence of disease, vaccination status, and age was tested using logistic regression with PROC LOGISTIC procedure. As all isolates were susceptible to linezolid and vancomycin, these antibiotics were excluded from the analysis.

Logistic regression was also used to analyze the influence of seven variables (sampling season, sample origin, and each animal’s sex, presence or absence of disease, vaccination status, and age) on the phenotypic expression of different virulence factors. As all isolates were negative for DNAse production, this virulence factor was excluded from the analysis. Moreover, multinomial logistic regression was used to analyze the influence of the same seven variables (sampling season, sample origin, and each animal’s sex, presence or absence of disease, vaccination status, and age) on the isolates’ MAR and V. indexes.

The correlation between the MAR and V. indexes was evaluated using Spearman’s rank correlation with PROC CORR. The same analysis was used to assess the correlation between the MAR index and the isolates’ ability to produce biofilm.

The relationship with resistance to different antibiotics was evaluated using Spearman’s rank correlation coefficient with PROC CORR while considering two levels of resistance—susceptible and resistant—with intermediate isolates being considered resistant for this analysis.

As suggested by Heinze and Dunkler [20], manual backward elimination with a *p* value criterion of 0.157 and without preceding univariable prefiltering was performed to reach the final models. In the final models, differences were considered significant when *p* < 0.05.

## 3. Results

A total of 32 dogs were enrolled in this study, including 16 animals sampled in the wet season and 16 animals sampled in the dry season. The sampled dogs belonged to the following breeds: German Shepherd (n = 8; 25%), Bullmastiff crossbreed (n = 3; 9%), Bullmastiff (n = 3; 9%), Rottweiler (n = 3; 9%), Pit Bull (n = 2; 6%), White Swiss Shepherd Dog (n = 2; 6%), Boerboel (n = 1; 3%), Poodle (n = 1; 3%), Chow Chow (n = 1; 3%), Labrador (n = 1; 3%), and undefined breed (n = 7; 22%). The animals’ age distribution ranged between 2 and 72 months of age (average of 12 months), and they were equally distributed regarding gender, with 6% being neutered. Considering vaccinations, 15 dogs (47%) had been vaccinated at least once, 7 (22%) dogs were parasitized by external parasites, and none had outdoor access. At the time of sampling, 50% (n= 16) of the dogs were healthy, and 50% (n = 16) presented clinical signs of disease (Supplementary Table S1).

It was possible to obtain presumptive staphylococci isolates from 26 (81%) of the sampled animals. An average of four isolates per dog sample were collected, with a total of 115 isolates. From these, 68 (59%) were obtained from samples collected during the dry season, and 47 (41%) were obtained from samples collected in the wet season. Moreover, 21 (24%) isolates were originated from oral samples, 31 (27%) from skin samples, and 56 (49%) from nasal samples.

### 3.1. Isolate Fingerprinting

The molecular profiles of the 115 isolates determined via amplification with the primer (GTG)_5_ showed several well-resolved fragments suitable for densitometric analysis and comparison. The reproducibility of the method was determined by calculating the average similarity value for all pairs of duplicates, which was found to be 81%. This value was used as the cutoff for defining clones.

Isolates from each animal with identical profiles at 81% or higher were considered clones, with only one isolate being selected from each clone for further characterization, resulting in a total of 40 isolates. However, 16 isolates were also retained for further characterization, as they corresponded to isolates with identical profiles but originated from different animals. As such, a total of 56 representative isolates were selected for further analysis, accounting for 48.7% of the total number of isolates obtained. The results from the isolates’ fingerprinting are presented in Supplementary Figure S1.

### 3.2. Isolate Identification

The 56 representative isolates selected after PCR fingerprinting were identified as follows: *Mammaliicoccus sciuri* (formerly *Staphylococcus sciuri*, n = 21; 38%), *Staphylococcus xylosus* (n = 17; 30%), *Staphylococcus equorum* (n = 7; 13%), *Mammaliicoccus vitulinus* (formerly *Staphylococcus vitulinus*, n = 4; 7%), *Mammaliicoccus lentus* (formerly *Staphylococcus lentus*, n = 3; 5%) and *Staphylococcus aureus* (n = 1; 2%). Three isolates were classified as *Staphylococcus* spp. (n = 3; 5%) (Supplementary Table S2).

### 3.3. Characterization of the Isolates’ Antimicrobial Resistance Profile

A high number of isolates (n = 48; 86%) were resistant to at least 1 of the 10 antimicrobials tested, and none of the isolates showed resistance to linezolid or vancomycin (Table 1). Methicillin-resistant (MRS) isolates were detected in all identified species (Table 2).

A total of 30% (n = 17) of the isolates were categorized as multidrug-resistant (MDR) (Table 2). Also, one *S. xylosus* isolate (2%) was considered extensively drug-resistant (XDR) [18].

The MAR index of the isolates under study ranged from 0 to 0.8, with a mean value of 0.2 (Table 2). These indexes differed significantly among the isolates collected in different seasons (*p* = 0.0018), with isolates from samples collected during the dry season presenting significantly higher scores. Isolates collected from dogs with signs of disease (*p* = 0.0014) and from older dogs (*p* = 0.0057) also presented higher MAR index values.

**Figure 1:**
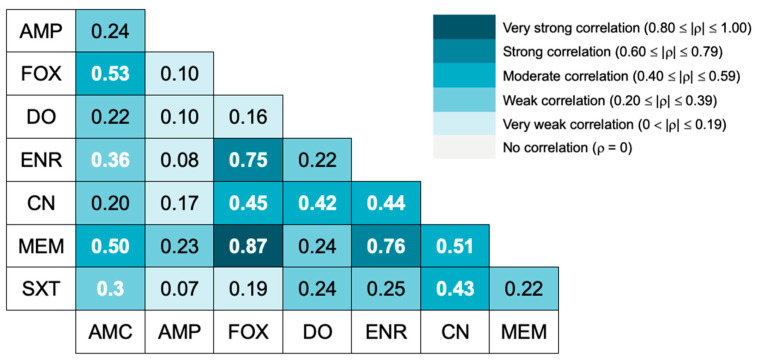
Spearman correlation between antimicrobial resistances to different antibiotics. A heat map shows the Spearman ⍴ value. White bold numbers indicate statistically significant correlation coefficients (*p* < 0.05). AMC = amoxicillin-clavulanate, AMP = ampicillin, FOX = cefoxitin, MEM = meropenem, DO = doxycycline, ENR = enrofloxacin, CN = gentamicin, SXT = trimethoprim/sulfamethoxazole.

The correlation between the isolates’ resistance to different antibiotics is presented in Figure 1. A quite strong correlation was only observed between meropenem and cefoxitin resistance. Strong resistance correlations were obtained between enrofloxacin and cefoxitin resistance and between enrofloxacin and meropenem resistance.

The impact of individual and environmental factors on the isolates’ resistance towards all antimicrobials tested was analyzed by multivariable logistic regression. The variables that presented statistically significant impacts on AMR (including odds ratios and *p* values) are summarized in Table 3.

Regarding resistance to amoxicillin-clavulanate, isolates from unvaccinated dogs were less likely (OR: 0.061) to be resistant to this antimicrobial than those from vaccinated dogs, and the same was observed for meropenem (OR: 0.142). Considering resistance to cefoxitin, isolates obtained from the dry season (OR: 5.772), sick animals (OR: 13.970), and young animals (OR:1.095) were more likely to be resistant to this antimicrobial than those from the wet season, healthy animals, or older animals, respectively. Regarding resistance to enrofloxacin, isolates obtained from female dogs were more likely to be resistant to this antimicrobial (OR: 5.014) than the isolates obtained from males. Also, considering resistance to meropenem, the isolates obtained from female dogs (OR:9.350) and sick animals (19.129) were more likely to be resistant to this antimicrobial.

### 3.4. Characterization of the Isolates’ Virulence Profile

Most of the isolates were able to produce biofilm (n = 52; 93%), and 46% (n = 26) were able to express lecithinase and gelatinase and 23% (n = 13) were able to produce hemolysins. None of the isolates was able to produce DNase.

The impact of individual and environmental factors on the isolates’ virulence profile was also analyzed through multivariable logistic regression. No significant differences were found between the isolates’ ability to form biofilm and the different factors tested. There was also no correlation (Spearman’s rank correlation) between biofilm production and the MAR index (*p* = 0.1810).

The V. index of the isolates varied between 0 and 0.8, with a mean value of 0.42 (Supplementary Table S3). These indexes were significantly influenced by the isolates’ species (*p* = 0.0009). *S. equorum* presented a higher V. index than *M. vitulinus* (*p* = 0.0257), while *M. sciuri* presented a higher V. index than *Staphylococcus* spp. (*p* = 0.0060), *M. vitulinus* (*p* < 0.0001), and *S. xylosus* (*p* = 0.0003).

No correlation (Spearman’s rank correlation) was found between the V. index and the MAR index (*p* = 0.6884).

The impact of the individual and environmental factors on the isolates’ ability to produce the tested virulence factors was also analyzed. The variables that presented statistically significant impacts on the expression of virulence factors are summarized in Table 4.

## 4. Discussion

Staphylococci are common inhabitants of the skin and mucosa of humans and animals, but they can also be responsible for serious public health problems [4]. Considering the One Health concept, which connects the human, animal, and environmental sectors, the dissemination of resistant bacteria across these sectors leads to an increased health risk for all [3]. As staphylococci are recognized as relevant in the spread of AMR, a phenomenon not fully addressed in LMI countries, especially in the veterinary sector, in this study, we focused on the isolation and characterization of staphylococci obtained from samples collected from the skin, oral, and nasal cavities of healthy and sick dogs from Angola.

The epidemiology of staphylococci in companion and production animals from Africa is not well documented [21,22]. According to a meta-analysis conducted by Ocloo et al. [22], *S. xylosus*, *S. pseudintermedius*, and *S. epidermidis* are the predominant staphylococcal species isolated across this continent. However, no articles referring to staphylococci with a veterinary origin in Angola were mentioned in their analysis. In fact, to the authors’ best knowledge, this study represents the first attempt to characterize the prevalence, resistance, and virulence profiles of canine staphylococci from Angolan veterinary settings.

Considering the 32 animals sampled in our study, 81% were positive for staphylococci, including isolates belonging to five different *Staphylococcus* or *Mammaliicoccus* species and three isolates only identified at the genus level. *M. sciuri* was the most prevalent species identified, which is consistent with previous reports by Chah et al. [23] and Siugzdaite and Gabinaitiene [24]. The presence of *M. sciuri* is concerning given its potential to carry antimicrobial and virulence genes, as documented by Couto et al. [25] and Stepanović et al. [26].

It was also possible to obtain *S. xylosus* isolates (30%). While typically regarded as a commensal *Staphylococcus* from the skin and mucosa of birds and mammals [27,28], opportunistic infections attributed to *S. xylosus* were already reported in humans and animals [29]. *S. equorum* (13%), *M. vitulinus* (7%), and *M. lentus* (5%) isolates were also obtained, with all being coagulase-negative staphylococci (CoNS) [30]. In our study, the CoNS prevalence was 98%, while the coagulase-positive staphylococci (CoPS) prevalence was only 2%, corresponding to one dog carrying *S. aureus* (2%). Low carriage rates of *S. aureus* by domestic animals have been previously reported [30,31]. In fact, the carriage of *S. aureus* in healthy animals is expected to be low, as colonization of dogs and cats by this bacterial species appears to be transient [32]. Also, our findings align with some studies which reported higher proportions of CoNS in dogs [33,34], contrary to the study performed by Qekwana et al. [35].

The isolates obtained presented high rates of resistance to antimicrobials commonly used in both human and veterinary medicine. This is particularly alarming as some of them exhibited resistance to last-choice antibiotics, such as meropenem [36]. Equally worrisome was the high percentage of MDR isolates (30%) observed in our study, which is in accordance with the study by Schmidt et al. [30]. This raises significant concerns, as these isolates may become opportunistic and lead to infections with limited treatment options. Additionally, it was possible to obtain one XDR isolate susceptible only to linezolid and vancomycin, which is particularly concerning as these are considered last-resort antibiotics that should not be used in veterinary medicine [37].

MRS, particularly MRSA, represent a serious problem for human and veterinary medicine, being linked to several difficult-to-treat infections [38]. The only *S. aureus* isolate identified in our study was obtained from a healthy dog, being classified as MRSA and MDR. Also, 30% of the CoNS isolates from different species were methicillin-resistant (MRCoNS). In comparison, Chah et al. [23] reported a lower prevalence of MRCoNS in dog samples, while Elnageh et al. [39] obtained similar results. These results are problematic, as MRS severely limit treatment options due to their resistance to common antibiotics [40].

Regarding the correlation between the isolates’ resistance to different antimicrobials, a strong correlation between cefoxitin and enrofloxacin resistance was found, as well as a moderate correlation between cefoxitin and gentamicin resistance. A study conducted by Adiguzel et al. [40] analyzed the in vitro cross-resistance patterns of MRCoNS isolates, reporting multiple cross-resistances with a wide variety of antimicrobials, including gentamicin and enrofloxacin, which corroborates our findings. The presence of cross-resistance in MRCoNS isolates significantly contributes to the lack of treatment options in cases of opportunistic infections [40].

The correlation between cefoxitin and meropenem resistance was the strongest correlation found in our study. Souza et al. previously reported cross-resistance between cefoxitin, oxacillin, and meropenem in *S. aureus* from cows [41]. Carbapenemase resistance is primarily mediated by mobile genetic elements containing beta-lactamase genes that also confer resistance to most penicillins and cephalosporins [42]. In our study, all meropenem-resistant isolates were also cefoxitin-resistant, and only one meropenem-resistant isolate was ampicillin-susceptible. Both the strong correlation detected and the high prevalence of meropenem resistance found were unexpected. Meropenem, along with other carbapenems, is known to have poor activity against MRSA and MRCoNS [43], and it is not available for veterinary use. A study by Elnageh et al. [39] on staphylococci from dogs and cats described an imipenem resistance rate of 34% in MRS isolates. Another study focusing on *S. aureus* isolates from cows also detected meropenem resistance in 56.25% of the isolates under study [41]. To the best of the authors’ knowledge, this is the first report of phenotypic resistance to meropenem in CoNS strains from dogs, reinforcing the importance of AMR surveillance in commensal isolates.

All isolates were susceptible to linezolid and vancomycin, aligning with the findings from Chah et al. [23] and Gandolfi-Decristophoris et al. [44]. This is a reassuring outcome, as vancomycin and linezolid are last-resort antibiotics that remain effective against MDR staphylococcal infections [45]. Despite this, surveillance studies should include these two compounds, as resistance to both linezolid and vancomycin has been previously documented in staphylococci from both animals and humans [22,39].

The MAR indexes varied significantly with the season, as isolates collected during the dry season exhibited higher MAR index scores. Seasonal changes can impact AMR rates due to variation in host susceptibility, environmental factors, changes in population behavior, and interactions among pathogens [46]. Animals presenting clinical signs of disease and older dogs also presented higher MAR indexes, potentially due to a compromised immune system.

The characterization of the isolates’ virulence profile revealed that CoNS species possessed the ability to express virulence traits contributing to their potential pathogenicity. Biofilm production enables bacteria to evade host defenses and impairs antibiotics’ action, contributing to the spread of AMR [47]. In our study, 93% of the isolates were capable of producing biofilms, which is in accordance with the work of Stepanović et al. [26]. Nevertheless, no correlation between biofilm production and the MAR index was found. The relationship between the MDR phenotype and biofilm-producing ability is a controversial matter, and there is still a lack of consensus among researchers [48].

DNAse, gelatinase, and lecithinase are enzymes that contribute to immune evasion and infection dissemination [26,47,49]. In this study, nearly half of the isolates were able to express both lecithinase and gelatinase, while 23% produced hemolysins. These are crucial virulence factors that contribute to cell damage and lysis [26], with hemolytic activity in CoNS species already being described [47,49,50].

The isolates presented a mean V. index value of 0.42, which indicates a potential higher risk of invading host defenses and causing disease. Specifically, *M. sciuri* presented a higher likelihood of producing virulence factors, presenting a higher V. index in comparison with the other species, probably due to their predominance in the samples collected for this study. Also, lecithinase production was higher in the isolates from samples collected during the wet season than in those obtained from samples collected in the dry season. This could hypothetically be attributed to variations in environmental factors, such as humidity, temperature, and microbial activity, which can influence the expression of virulence [51]. The bacterial species significantly influenced the isolates’ ability to express gelatinase and lecithinase, with *M. sciuri* presenting a higher likelihood of producing those virulence factors than the other bacterial species identified in our study. As previously mentioned, this could be attributed to a potential sampling bias.

Considering that CoNS species predominantly colonize the skin, the presence of breaches in the skin epithelium, coupled with the expression of virulence factors by these strains, may promote bacterial adherence and invasion and, consequently, infection development [52].

This study presents some limitations, primarily related to sample collection, which was directly influenced by the animals presented for consultation at the veterinary clinic that participated in this study. Additionally, it was difficult to collect complete information for the sampled animals and proceed with their follow-ups, and for three cases, the final diagnosis could not be obtained, as detailed in Table S1. To improve risk assessment, it would also be relevant to collect information on resistant bacteria carriage by pet owners. Lastly, future studies should focus on the genomic characterization of the isolates to better understand the resistance determinants present in the identified strains.

## 5. Conclusions

Our results show that the skin and mucosa of the dogs were predominantly colonized by coagulase-negative staphylococci, and independently of their health status, these animals can carry antimicrobial-resistant staphylococci, including MDR strains and isolates exhibiting resistance to last-resort antimicrobials such as meropenem. The threat posed by these strains, which were also able to express several virulence factors that could increase their pathogenic potential, is amplified by the scarcity of antimicrobial treatment options for infection control.

Considering the interconnectedness of animal and human health and the absence of a national plan for AMR control in Angola, our results underscore the necessity of a better understanding of the prevalence and characterization of staphylococci from companion animals and their owners in this country. Further studies are encouraged, aiming to assess the genetic determinants associated with AMR and virulence, with emphasis on MDR and XDR isolates, contributing to a deeper understanding of the risks associated with AMR in this context.

## Figures and Tables

**Table 1:** Antimicrobial susceptibility profile of the staphylococci and mammaliicocci under study.

Antimicrobial Class	Antimicrobial Compound	Concentration (μg)	Number of Isolates (n = x (%))	
			Susceptible (S)	Resistant (R)
Beta-Lactams	Amoxicillin-Clavulanate (AMC)	30	47 (84)	9 (16)
	Ampicillin (AMP)	10	13 (23)	43 (77)
	Cefoxitin (FOX)	30	38 (68)	18 (32)
	Meropenem (MEM)	10	41 (73)	15 (27)
Tetracyclines	Doxycycline (DO)	30	43 (77)	13 (23)
Fluoroquinolones	Enrofloxacin (ENR)	5	44 (79)	12 (21)
Aminoglycosides	Gentamicin (CN)	10	51 (91)	5 (9)
Oxazolidinones	Linezolid (LZD)	30	56 (100)	0 (0)
Folate Antagonists	Trimethoprim/Sulfamethoxazole (SXT)	25	55 (98)	1 (2)
Glycopeptides	Vancomycin (VA)	30	56 (100)	0 (0)

**Table 2:** Number of multidrug-resistant (MDR) isolates, range of the multiple antimicrobial resistance (MAR) index, and number of methicillin resistant isolates by bacterial species.

Species	Total N° of Isolates	N° of MDR Isolates	MAR Index Range	Mean MAR Index	N° of Methicillin Resistant Isolates
*M. sciuri*	21	5	0–0.5	0.17	4
*S. xylosus*	17	6	0.1–0.8	0.27	7
*S. equorum*	7	0	0–0.1	0.04	1
*M. vitulinus*	4	2	0–0.4	0.25	2
*Staphylococcus* spp.	3	1	0.1–0.3	0.17	1
*M. lentus*	3	2	0.1–0.6	0.33	2
*S. aureus*	1	1	0.7	0.7	1
Total	56	17	0–0.8	0.2	18

**Table 3:** Variables identified as risk factors for the presence of antimicrobial-resistant bacteria in the sampled dogs.

Antimicrobial	Variable	OR	95% CI	*p* Value
Amoxicillin-clavulanate	Vaccination (without vaccines vs. with at least one dose)	0.061	0.006–0.569	0.0142
Cefoxitin	Sampling season (dry vs. wet)	5.772	1.254–26.581	0.0244
	Presence of disease (sick vs. healthy)	13.970	2.644–73.805	0.0019
	Age (months)	1.095	1.018–1.177	0.0144
Enrofloxacin	Sex (female vs. male)	5.014	1.148–21.900	0.0321
Meropenem	Sex (female vs. male)	9.350	1.541–56.733	0.0151
	Presence of disease (sick vs. healthy)	19.129	2.539–144.096	0.0042
	Vaccination (without vaccines vs. with at least one dose)	0.142	0.023–0.866	0.0343

Abbreviations: OR = odds ratio; CI = confidence interval.

**Table 4:** Variables identified as risk factors for the production of virulence factors by the dog isolates under study.

	Variable		OR	95% CI	*p* Value
Hemolysins	Sex		4.200	1.106–15.949	0.0350
Lecithinase	Season (dry vs. wet)		0.113	0.016–0.777	0.0266
	Bacterial species	*S. equorum* vs. *M. sciuri*	0.014	0.001–0.205	0.0017
		*M. sciuri* vs. *M. vitulinus*	37.905	1.819–789.923	0.0190
		*M. sciuri* vs. *S. xylosus*	32.381	4.432–236.598	0.0006
Gelatinase		*S. equorum* vs. *M. sciuri*	0.009	<0.001–0.183	0.0022
		*M. sciuri* vs. *M. vitulinus*	96.785	3.791–>999.999	0.0057
		*M. sciuri* vs. *S. xylosus*	80.415	7.163–902.790	0.0004

Abbreviations: OR = odds ratio; CI = confidence interval.

## Data Availability

The data presented in this study are available on request from the corresponding author. The data are not publicly available due to privacy reasons.

## Supplementary Materials

The following supporting information can be downloaded at https://www.mdpi.com/article/10.3390/ani15071043/s1. Table S1: Sampling data. Figure S1: Similarity of 56 isolates calculated by Pearson correlation coefficient and clustering by UPGMA based on the (GTG)_5_ profiles of BioNumerics^®^ 6.6. Additionally, the data for eight antibiotics [amoxicillin-clavulanate (AMC), ampicillin (AMP), cefoxitin (FOX), doxycycline (DO), enrofloxacin (ENR), gentamicin (CN), meropenem (MEM), and trimethoprim/sulfamethoxazole (SXT)] are provided for each isolate, with a black square indicating resistance and a gray square indicating susceptibility. Similarly, the presence of four virulence factors (hemolysins, biofilm, lecithinase, and gelatinase) is shown, with a black square noting their presence and a gray square indicating that they were not detected. The bacterial species identification is also presented. Table S2: Identification protocols and VITEK identification of the staphylococci isolates. Table S3: Resistance and virulence profiles of the isolates (n = 56). Legend: biofilm production (B); DNAse activity (D); lecithinase activity (L); hemolysin production (H); gelatinase activity (G); amoxicillin-clavulanate (AMC); ampicillin (AMP); cefoxitin (FOX); doxycycline (DO); enrofloxacin (ENR); gentamicin (CN); meropenem (MEM); trimethoprim/sulfamethoxazole (SXT); not found (-).
