# Supplementary material for: Insights into Molecular Profiles, Resistance Patterns, and Virulence Traits of Staphylococci from Companion Dogs in Angola
Source: Animals (Basel). 2025 Apr 4;15(7):1043. doi: 10.3390/ani15071043 (PMC11987833; doi:10.3390/ani15071043)
Supplement: Supplementary file 1 [file animals-15-01043-s001.zip › animals-3502949-supplementary.pdf]

Table S1: Sampling data.

Dog	Period	Date	Age (months)	Sex	Breed	Neutered/spayed or intact status	Outdoor acess	Presence or absence of disease	Presumptive diagnosis	Vaccination schedule	Deworming /parasite control
**1**	Wet	17/04/22	11	F	German Shepherd	Intact	No	No		Primodog®, Tetradog®, Tetradog® and Hexadog®	Yes
**2**	Wet	17/04/22	72	F	Chow-chow	Intact	No	No		Hexadog®	Yes
**3**	Wet	17/04/22	24	M	NDB	Neutered	No	No		rimodog®, Tetradog®, Tetradog® and Hexadog®	Yes
**4**	Wet	19/04/22	2	M	Poodle	Intact	No	Yes	Parvovirus	-	No
**5**	Wet	19/04/22	5	M	Pit Bull	Intact	No	No		Primodog®, Tetradog®, Tetradog® and Hexadog®	Yes
**6**	Wet	19/04/22	3	M	German Shepherd	Intact	No	Yes	Parvovirus	Tetradog®	Yes
**7**	Wet	19/04/22	4	F	German Shepherd	Intact	No	No		Tetradog® and Tetradog®	Yes
**8**	Wet	21/04/22	3	M	Bullmastiff	Intact	No	Yes	Parvovirus	-	No
**9**	Wet	21/04/22	72	F	Rottweiler	Spayed	No	Yes	Non defined	Primodog®, Tetradog®, Tetradog®, Hexadog®, Hexadog® and Hexadog®	No
**10**	Wet	20/04/2022	20	M	Bullmastiff	Intact	No	No		Tetradog®, Tetradog®,	No

Dog	Period	Date	Age (months)	Sex	Breed	Neutered/spayed or intact status	Outdoor acess	Presence or absence of disease	Presumptive diagnosis	Vaccination schedule	Deworming /parasite control
										Rabies and Hexadog®	
**11**	Wet	26/04/22	3	M	German Shepherd	Intact	No	Yes	Parvovirus	-	Yes
**12**	Wet	28/04/22	4	F	NDB	Intact	No	No		Tetradog®	Yes
**13**	Wet	21/04/22	4	M	Bullmastiff cross	Intact	No	Yes	Ehrlichiosis	-	Yes
**14**	Wet	03/05/22	3	F	Rottweiler	Intact	No	Yes	Parvovirus	-	Yes
**15**	Wet	04/05/22	4	M	Boerboel	Intact	No	Yes	Parvovirus	-	No
**16**	Wet	05/05/22	Non defined	F	Pit Bull	Intact	No	No		-	No
**17**	Dry	08/06/22	12	F	NDB	Intact	No	Yes	Ehrlichiosis	Primodog®, Tetradog®	No
**18**	Dry	08/06/22	24	F	Rottweiler	Intact	No	No		Primodog®, Tetradog®, Tetradog® and Rabies	No
**19**	Dry	14/06/22	6	F	Bullmastiff	Intact	No	Yes	Ehrlichiosis	-	No
**20**	Dry	14/06/22	2	M	German Shepherd	Intact	No	No		-	No
**21**	Dry	14/06/22	24	F	Bullmastiff	Intact	No	No		-	No
**22**	Dry	15/06/22	4	M	Bullmastiff cross	Intact	No	No		Primodog®, Tetradog®, Tetradog® and Hexadog®	Yes
**23**	Dry	23/06/22	3	M	NDB	Intact	No	No		-	No
**24**	Dry	23/06/22	3	F	NDB	Intact	No	No		-	No
**25**	Dry	23/06/22	6	M	White Swiss	Intact	No	No		-	No

Dog	Period	Date	Age (months)	Sex	Breed	Neutered/spayed or intact status	Outdoor acess	Presence or absence of disease	Presumptive diagnosis	Vaccination schedule	Deworming /parasite control
					Shepherd Dog						
**26**	Dry	23/06/22	6	F	White Swiss Sheperd dog	Intact	No	Yes	Ehrlichiosis	-	No
**27**	Dry	28/06/22	14	F	German Shepherd	Intact	No	No		Primodog®, Tetradog®, Tetradog® and Hexadog®	No
**28**	Dry	29/06/22	4	F	NDB	Intact	No	Yes	Parvovirus	-	Yes
**29**	Dry	29/06/22	7	M	German Shepherd	Intact	No	Yes	Babesiosis	Primodog®	No
**30**	Dry	07/07/22	3	F	Labrador Retriever	Intact	No	Yes	Parvovirus	-	No
**31**	Dry	20/07/22	4	M	German Shepherd	Intact	No	Yes	ND	Hexadog®	Yes
**32**	Dry	28/07/22	4	M	NDB	Intact	No	Yes	ND	-	No

Legend: F- Female; M- Male; ND- Non defined.

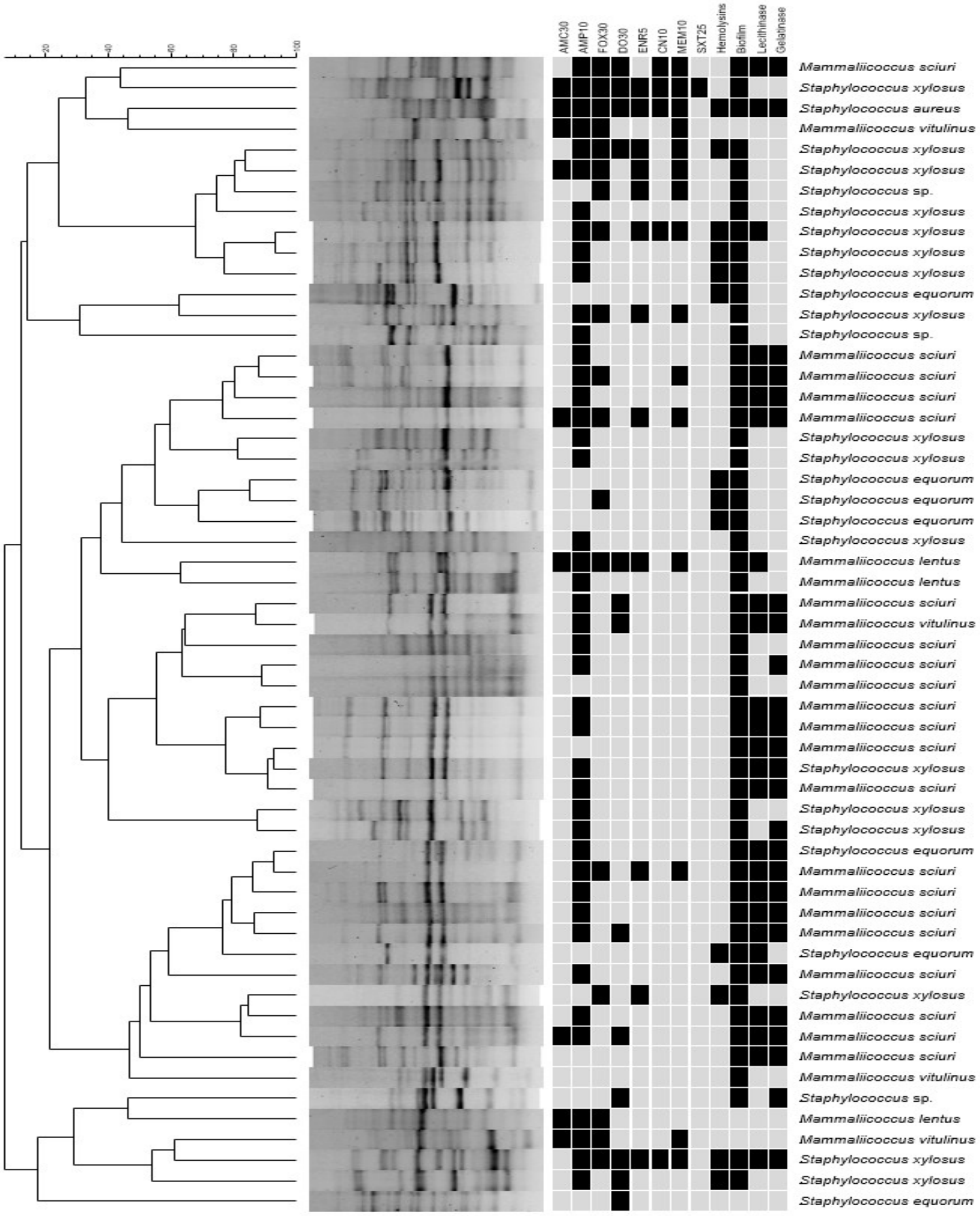

Figure S1: Similarity of 56 isolates calculated by Pearson correlation coefficient and clustering by UPGMA based on the (GTG)5 profiles by BioNumerics® 6.6. Additionally, the data for eight antibiotics [Amoxicillin-clavulanate (AMC), Ampicillin (AMP), Cefoxitin (FOX), Doxycycline (DO), Enrofloxacin (ENR), Gentamicin (CN), Meropenem (MEM) and Trimethoprim/Sulfamethoxazole (SXT)] is provided for each isolate, with a black square indicating resistance and a grey square indicating susceptibility. Similarly, the presence of four virulence factors (Hemolysins, Biofilm, Lecithinase and Gelatinase) is shown, with a black square enoting their presence and a grey square indicating that they were not detected. The bacterial species identification is also presented.

Table S2: Identification protocols and VITEK identification of the staphylococci isolates.

Animal	Isolate	GRAM	Manitol	Catalase	Coagulase	Hemolysis	Identification
**1**	1N4	Pos	Pos	Pos	Neg	Neg	*M. sciuri*
	1P4	Pos	Pos	Pos	Neg	Neg	*S. equorum*
**2**	2B2	Pos	Pos	Pos	Neg	Neg	*M. sciuri*
	2B4	Pos	Pos	Pos	Neg	Neg	*M. sciuri*
**3**	3N1	Pos	Pos	Pos	Neg	Neg	*M. sciuri*
	3N2	Pos	Pos	Pos	Neg	Neg	*M. sciuri*
**4**	4N1	Pos	Pos	Pos	Neg	Neg	*M. sciuri*
	4N2	Pos	Pos	Pos	Neg	Neg	*S. sciuri*
**5**	5B1	Pos	Pos	Pos	Neg	Neg	*S. equorum*
	5B2	Pos	Pos	Pos	Neg	Neg	*S. equorum*
**6**	6B2	Pos	Pos	Pos	Neg	Neg	*S. equorum*
	6N1	Pos	Pos	Pos	Neg	Neg	*M. sciuri*
**7**	7P1	Pos	Pos	Pos	Neg	Neg	*S. equorum*
**8**	8B1	Pos	Pos	Pos	Neg	Neg	*S. equorum*
	8N2	Pos	Pos	Pos	Neg	Neg	*M. sciuri*
**9**	9N1	Pos	Pos	Pos	Neg	Neg	*S. equorum*
**10**	10N2	Pos	Pos	Pos	Neg	Neg	*M. sciuri*
**11**	11B1	Pos	Pos	Pos	Neg	Neg	*S. xylosus*
	11N2	Pos	Pos	Pos	Neg	Neg	*M. vitulinus*
	11N4	Pos	Pos	Pos	Neg	Neg	*M. vitulinus*
	11P4	Pos	Pos	Pos	Neg	Neg	*Staph*. sp
**12**	12N1	Pos	Pos	Pos	Neg	Neg	*S. xylosus*
	12P1	Pos	Pos	Pos	Neg	Neg	*S. xylosus*
**13**	13N4	Pos	Pos	Pos	Neg	Neg	*S. xylosus*
**14**	14B3	Pos	Pos	Pos	Neg	Neg	*M. lentus*
**19**	19P2	Pos	Pos	Pos	Neg	Neg	*S. xylosus*
	19B1	Pos	Pos	Pos	Neg	Neg	*S. xylosus*
**20**	20N1	Pos	Pos	Pos	Neg	Neg	*S. xylosus*
**21**	21B3	Pos	Pos	Pos	Neg	Neg	*M. sciuri*
**22**	22N3	Pos	Pos	Pos	Neg	Neg	*Staph*. sp
	22P3	Pos	Pos	Pos	Neg	Neg	*S. xylosus*
	22B2	Pos	Pos	Pos	Neg	Neg	*S. xylosus*
**23**	23N3	Pos	Pos	Pos	Neg	Neg	*S. xylosus*
**24**	24B1	Pos	Pos	Pos	Neg	Neg	*M. lentus*
	24N1	Pos	Pos	Pos	Neg	Neg	*M. sciuri*
	24N2	Pos	Pos	Pos	Neg	Neg	*M. sciuri*
	24N4	Pos	Pos	Pos	Neg	Neg	*M. sciuri*
**25**	25N1	Pos	Pos	Pos	Neg	Neg	*M. sciuri*
	25N2	Pos	Pos	Pos	Neg	Neg	*M. sciuri*
	25P1	Pos	Pos	Pos	Neg	Neg	*M. sciuri*
**27**	27N1	Pos	Pos	Pos	Pos	Neg	*S. aureus*
	27N3	Pos	Pos	Pos	Neg	Neg	*M. sciuri*
	27N4	Pos	Pos	Pos	Neg	Neg	*M. sciuri*
**28**	28N1	Pos	Pos	Pos	Neg	Neg	*S. xylosus*
	28N2	Pos	Pos	Pos	Neg	Neg	*Staph*. sp
	28N4	Pos	Pos	Pos	Neg	Neg	*S. xylosus*
**29**	29P1	Pos	Pos	Pos	Neg	Neg	*M. vitulinus*
	29B1	Pos	Pos	Pos	Neg	Neg	*S. xylosus*
	29B2	Pos	Pos	Pos	Neg	Neg	*M. vitulinus*
	29B4	Pos	Pos	Pos	Neg	Neg	*M. lentus*
	29N4	Pos	Pos	Pos	Neg	Neg	*M. sciuri*
**30**	30N1	Pos	Pos	Pos	Neg	Neg	*S. xylosus*
	30N4	Pos	Pos	Pos	Neg	Neg	*S. xylosus*
**31**	31P2	Pos	Pos	Pos	Neg	Neg	*S.xylosus*
	31N3	Pos	Pos	Pos	Neg	Neg	*S. xylosus*

Legend: Pos- positive; Neg- Negative.

Table S3: Resistance and Virulence profiles of the isolates (n=56); Legend: Biofilm production (B); DNAse activity (D); Lecithinase activity (L); Hemolysin production (H); Gelatinase activity (G); Amoxicillin-clavulanate (AMC); Ampicillin (AMP); Cefoxitin (FOX); Doxycycline (DO); Enrofloxacin (ENR); Gentamicin (CN); Meropenem (MEM); Trimethoprim/Sulfamethoxazole (SXT); Not Found (-).

Isolates	Species	Resistance Profile		Virulence Profile	
		Resistant	MAR Index	Positive Results	Virulence index
1N4	*M. sciuri*	AMP	0.1	B; L; G	0.6
1P4	*S. equorum*	-	0	H; B	0.4
2B2	*M. sciuri*	AMP; FOX; MEM	0.3	B; L; G	0.6
2B4	*M. sciuri*	AMC; AMP; FOX; ENR; MEM	0.5	B; L; G	0.6
3N1	*M. sciuri*	AMP	0.1	B; L; G	0.6
3N2	*M. sciuri*	AMP	0.1	B; L; G	0.6
4N1	*M. sciuri*	AMP	0.1	B; L; G	0.6
4N2	*M. sciuri*	-	0	B; L; G	0.6
5B1	*S. equorum*	-	0	H; B	0.4
5B2	*S. equorum*	-	0	H; B; L;	0.6
6B2	*S. equorum*	AMP	0.1	B; L; G	0.6
6N1	*M. sciuri*	AMP	0.1	B; L; G	0.6
7P1	*S. equorum*	-	0	H; B	0.4
8B1	*S. equorum*	DO	0.1	-	0.4
8N2	*M. sciuri*	AMP; FOX; ENR; MEM	0.4	B; L; G	0.6
9N1	*S. equorum*	FOX	0.1	H; B	0.4
10N2	*M. sciuri*	AMP; DO	0.2	B; L; G	0.6
11B1	*S. xylosus*	AMP	0.1	B; L; G	0.6
11N2	*M. vitulinus*	-	0	B	0.2
11N4	*M. vitulinus*	AMP; DO	0.2	B; L; G	0.6
11P4	*Staph*. sp	AMP	0.1	B	0.2
12N1	*S. xylosus*	AMP; FOX; ENR; MEM	0.4	B	0.2
12P1	*S. xylosus*	AMP	0.1	B	0.2
13N4	*S. xylosus*	AMP	0.1	B	0.2
14B3	*M. lentus*	AMC; AMP; FOX; DO;ENR;MEM	0.6	B; L	0.4
19P2	*S. xylosus*	AMP	0.1	H; B;	0.4
19B1	*S. xylosus*	AMP; FOX; ENR; CN; MEM	0.5	H; B; L	0.6
20N1	*S. xylosus*	AMP	0.1	H; B	0.4
21B3	*M. sciuri*	-	0	B; L; G	0.6
22N3	*Staph*. sp	DO	0.1	B; G	0.4
22P3	*S. xylosus*	AMP	0.1	B	0.2
22B2	*S. xylosus*	AMP	0.1	B; G	0.4
23P2	*S. xylosus*	FOX; ENR	0.2	H; B	0.4
23N3	*M. sciuri*	AMP	0.1	B; G	0.4
24B1	*M. lentus*	AMP	0.1	B	0.2
24N1	*M. sciuri*	-	0	B	0.2
24N2	*M. sciuri*	AMP	0.1	B; L; G	0.6
24N4	*M. sciuri*	AMP	0.1	B	0.2
25N1	*M. sciuri*	AMP	0.1	B; L; G	0.6
25N2	*M. sciuri*	AMP	0.1	B; L; G	0.6
25P1	*M. sciuri*	AMP	0.1	B; L; G	0.6
27N1	*S. aureus*	AMC; AMP; FOX; DO; ENR;CN; MEM	0.7	H; B; L; G	0.8
27N3	*M. sciuri*	AMC; AMP; DO	0.3	B; L; G	0.6

Isolates	Species	Resistance Profile		Virulence Profile	
		Resistant	MAR Index	Positive Results	Virulence index
27N4	*M. sciuri*	AMP; DO	0.2	B; L; G	0.6
28N1	*S. xylosus*	AMP	0.1	B	0.2
28N2	*Staph*. sp	FOX; ENR; MEM	0.3	B	0.2
28N4	*S. xylosus*	AMP; FOX; DO; ENR; MEM	0.5	H; B	0.4
29P1	*M. vitulinus*	AMC; AMP; FOX; MEM	0.4	-	0
29B1	*S. xylosus*	AMP	0.1	B	0.2
29B2	*M. vitulinus*	AMC; AMP; FOX; MEM	0.4	-	0
29B4	*M. lentus*	AMC; AMP; FOX;	0.3	-	0
29N4	*M. sciuri*	AMP; FOX; DO; CN; MEM	0.5	B; L; G	0.6
30N1	*S. xylosus*	AMP; DO	0.2	H; B	0.4
30N4	*S. xylosus*	AMP; FOX; DO; ENR; CN; MEM	0.6	H; B; L; G	0.8
31P2	*S. xylosus*	AMC; AMP; FOX; DO; ENR; CN; MEM; SXT	0.8	B	0.2
31N3	*S. xylosus*	AMC; AMP; FOX; ENR; MEM	0.5	B	0.2
